# Special features of RAD Sequencing data: implications for genotyping

**DOI:** 10.1111/mec.12084

**Published:** 2012-10-30

**Authors:** John W Davey, Timothée Cezard, Pablo Fuentes-Utrilla, Cathlene Eland, Karim Gharbi, Mark L Blaxter

**Affiliations:** * Institute of Evolutionary Biology, School of Biological Sciences, University of EdinburghWest Mains Road, Edinburgh, EH9 3JT, UK; † The GenePool, Ashworth Laboratories, University of EdinburghWest Mains Road, Edinburgh, EH9 3JT, UK

**Keywords:** contig assembly, genotyping by sequencing, population genetics, RAD Sequencing, restriction enzymes

## Abstract

Restriction site-associated DNA Sequencing (RAD-Seq) is an economical and efficient method for SNP discovery and genotyping. As with other sequencing-by-synthesis methods, RAD-Seq produces stochastic count data and requires sensitive analysis to develop or genotype markers accurately. We show that there are several sources of bias specific to RAD-Seq that are not explicitly addressed by current genotyping tools, namely restriction fragment bias, restriction site heterozygosity and PCR GC content bias. We explore the performance of existing analysis tools given these biases and discuss approaches to limiting or handling biases in RAD-Seq data. While these biases need to be taken seriously, we believe RAD loci affected by them can be excluded or processed with relative ease in most cases and that most RAD loci will be accurately genotyped by existing tools.

## Introduction

The use of high throughput sequencing-by-synthesis technologies for ecology and conservation depends on accurate inference of biological signal from technical noise. Individual genotypes and population allele frequencies must be inferred from raw sequence data, preferably at low cost and with low sequencing and analytical effort. While it is now possible to generate sequence data from entire genomes at relatively low cost, the sequencing-by-synthesis process introduces noise from a number of novel sources and reveals existing sources of noise that were previously undetected by less sensitive technology, making the path from raw sequence reads to biological information far from straightforward. In recent years, many sources of noise in high throughput DNA and RNA sequencing data have been identified and either mitigated during library preparation or corrected during analysis (Aird *et al*. 2011; Quince *et al*. 2011, Meacham *et al*. 2011; Quail *et al*. 2008). However, methods appropriate for one sequencing method are not necessarily appropriate for others, and new library preparation methods may produce novel sources of noise.

Restriction site-associated DNA sequencing (RAD-Seq; Miller *et al*. 2007; Baird *et al*. 2008; Davey & Blaxter 2011) is a method for SNP discovery and genotyping using sequencing-by-synthesis. It is one of a number of reduced representation methods that sample a shared set of sites across the genome in many individuals or pools, making population-scale sequencing possible at a fraction of the cost of whole genome sequencing (Davey *et al*. 2011). RAD-Seq is suitable for fine-scale linkage mapping (Amores *et al*. 2011; Chutimanitsakun *et al*. 2011; Baxter *et al*. 2011), phylogenetics and phylogeography (Rubin *et al*. 2012; Nadeau *et al*. 2012, Emerson *et al*. 2010), genome scaffolding (Catchen *et al*. 2011; Heliconius Genome Consortium 2012) and population genetics (Andersen *et al*. 2012; Hohenlohe *et al*. 2012). RAD-Seq has also been used to generate large SNP data sets for many species, most recently in salmon (Houston *et al*. 2012), cutthroat and rainbow trout (Amish *et al*. 2012), artichoke (Scaglione *et al*. 2012), guppy (Willing *et al*. 2011) and eggplant (Barchi *et al*. 2011).

The RAD-Seq method has been well documented elsewhere (Baird *et al*. 2008; Etter *et al*. 2011a). Briefly, genomic DNA from multiple samples of interest is digested with a chosen restriction enzyme, and adapters that contain sample-specific barcodes and end with an overhang matching the restriction enyzme's cut site are ligated to the digested restriction fragments. Adapter-ligated restriction fragments are sheared to a size suitable for Illumina sequencing (typically 300–700 bp), and sheared fragments containing restriction site overhangs are amplified using polymerase chain reaction (PCR) and sequenced, typically using Illumina sequencing-by-synthesis.

RAD-Seq reads can be aligned to reference genomes and genotyped using standard tools designed for whole genome sequencing data (Nielsen *et al*. 2011), including aligners such as BWA (Li & Durbin 2009) and Stampy (Lunter & Goodson 2011), and genotypers such as those built into the Genome Analysis Tool Kit (GATK; DePristo *et al*. 2011) and SAMtools (Li 2011). RAD-Seq can also be used de novo, generating large marker sets where no reference genome is available. Several tools have been developed to produce RAD marker sets de novo, including Stacks (Catchen *et al*. 2011), RaPiD (Willing *et al*. 2011) and RADtools (Baxter *et al*. 2011).

RAD-Seq projects typically produce thousands to tens of thousands of markers, several orders of magnitude greater than is possible with traditional technologies such as microsatellites or AFLPs, at a fraction of the labour cost. However, separating high-quality markers from sequencing noise is challenging. Manual validation of such large marker sets is impractical, and the accuracy of automatic analysis tools is not yet clear. Unfortunately, because RAD-Seq has considerable benefits to researchers working with non-model species, the vast majority of publicly available RAD-Seq data are derived from populations with no reference genome or sequence variation information, making it difficult to validate RAD-Seq marker sets in any depth.

Typically, RAD-Seq analysis proceeds by applying quality thresholds or likelihood ratio tests at multiple levels (for example, raw sequence, mapping and genotyping), and by testing for expected biological patterns. For example, for linkage mapping, markers can be removed if they are missing in multiple individuals or have segregation distortion (Amores *et al*. 2011; Miller *et al*. 2011); for population studies, repetitive regions and duplicates can be screened by filtering by read coverage or by testing patterns of heterozygosity (Hohenlohe *et al*. 2011). Marker sets can be validated using laboratory methods such as PCR (e.g. Scaglione *et al*. 2012) or SNP chips, but as comprehensive validation of tens of thousands of markers remains expensive and labour-intensive, it would be valuable to improve bioinformatic filtering to reduce the cost of laboratory validation.

Illumina sequencing of short (<1 kb) fragments involves sequencing one (read 1, single end) or both (reads 1 and 2, paired end) ends of each fragment, typically producing reads 100 bp long. RAD markers can be developed using only single end Illumina sequencing, identifying single nucleotide polymorphisms (SNPs) and insertions or deletions (indels) in read 1 sequences. However, paired-end sequencing can also be used for RAD-Seq, a technique that has several novel implications compared with paired-end sequencing of genomic DNA and that makes RAD-Seq particularly attractive for de novo studies. First, read 2 sequences up- or downstream of a particular restriction site can be assembled into 300- to 600-bp RAD contigs (Etter *et al*. 2011b; Willing *et al*. 2011), which can be used to investigate synteny and gene content in otherwise unsequenced genomes (Baxter *et al*. 2011). Second, read 2 sequences have been used to attempt to remove PCR duplicates from RAD-Seq data (Baxter *et al*. 2011), with the aim of reducing GC bias known to be introduced by PCR (Benjamini & Speed 2012; Aird *et al*. 2011, Quail *et al*. 2008).

While RAD-Seq can be used for reference-based approaches, several methods with simpler library preparation protocols have been developed (e.g. Andolfatto *et al*. 2011; Elshire *et al*. 2011; Wang *et al*. 2012), which may be preferable for study of laboratory crosses or where a high-quality reference sequence is available (Davey *et al*. 2011), as this allows imputation of genotypes in the face of missing data. In theory, RAD-Seq develops markers more robustly than these related methods and so is more suitable for de novo analyses of wild populations, where little information about the source populations is known and so imputation of missing data is very difficult. However, no empirical study of technical variation in RAD-Seq data has been published to date. While RAD-Seq is in principle unbiased with respect to many population genetics statistics and so may avoid known issues of ascertainment bias in marker sets (Helyar *et al*. 2011), in practice there has been no detailed analysis of noise in RAD-Seq data, and it may be that commonly used quality thresholds and post hoc tests are unsuitable. This may mean researchers are discarding potentially useful markers, retaining inaccurate markers or incorrectly genotyping real markers.

We therefore set out to investigate the characteristics of RAD-Seq data, to validate existing analysis techniques and propose improvements where appropriate. In the process, we identified several sources of sequencing variation unique to RAD-Seq, above and beyond those found in other types of sequencing-by-synthesis data. These sources of variation have implications for genotyping of RAD markers. We also investigated methods for RAD contig assembly. Multiple assemblers have been used to generate RAD contigs, including Velvet (Catchen *et al*. 2011; Etter *et al*. 2011b), VelvetOptimiser (Baxter *et al*. 2011; Houston *et al*. 2012) and LOCASopt (Willing *et al*. 2011). However, to date, there has been no comparison of the performance of existing assemblers on heterozygous RAD paired-end data where reference sequences are available. We hope this work will bring clarity to the process of generating RAD-Seq data and enable thorough analysis of both simple and complex studies.

## Materials and methods

### *Caenorhabditis elegans* library preparation

*Caenorhabditis elegans* (N2 strain) worms were grown in agar plates as per the study by Lewis & Fleming 1995. Nearly starved worms were washed from plates, concentrated using sucrose flotation (Johnstone 1999) and stored at −80 °C. Genomic DNA (gDNA) was isolated from frozen worms using the DNeasy Blood and Tissue Kit (QIAGEN).

PstI-digested RAD libraries were prepared following the study by Baird *et al*. (2008), using 20 units of PstI-HF (New England BioLabs) to digest 1 μg gDNA per sample. Digested DNA was ligated to P1 barcoded adapters using four different barcodes for each of six libraries (total 4 μg gDNA per library). Ligations were sheared to a target peak of 400 bp using a Covaris S2 sonicator. To remove adapter dimers, libraries were purified with Agencourt AMPure XP (Beckman Coulter) magnetic beads after P2 adapter ligation with a volume DNA/beads ratio of 1:0.8. The six libraries were PCR amplified with a range of cycles (14x, 16x, 18x, 20x, 22x and 24x). PCR-enriched libraries were purified with AMPure XP beads, normalized to 10nM and pooled together for sequencing on one lane of an Illumina HiSeq 2000 flowcell (101-bp paired-end reads). Read counts are provided in Table S1, Supporting Information. Methods for plasmid shearing and variable shearing RAD libraries for Figs S2 and S3 are provided in Additional Methods (Supporting Information).

### *Caenorhabditis elegans* sequence analysis

To examine the relationship between restriction fragment length and read depth, PstI sites in the *C. elegans* reference genome and expected PstI restriction fragment sequences were identified (genome version WS229, downloaded from http://www.wormbase.org). Read pairs from *C. elegans* PstI RAD libraries with both reads perfectly matching one and only one PstI restriction fragment end (<1 kb) were retained. RAD loci with nonunique read 1 (96 bp) sequences or no coverage were excluded.

Normalized read counts shown in Fig. 4 were generated by calculating the proportion of reads for each sample compared with total reads for each library and adjusting each locus read count for each sample by this proportion. The same process was used to generate normalized sheared fragment counts. Table S1 lists counts and proportions for reads and fragments.

### Developing a validated set of Heliconius RAD loci

Heliconius PstI RAD samples publicly available as part of European Nucleotide Archive project ERP000993 were aligned to the *Heliconius melpomene* reference genome version 1.1 (available from Ensembl Genomes at http://metazoa.ensembl.org/Heliconius_melpomene) using Stampy v1.0.17 with parameters insertsize = 500, insertsd = 100, on the Edinburgh Compute and Data Facility compute cluster (ECDF, http://www.ecdf.ed.ac.uk/), partially supported by the eDIKT initiative (http://www.edikt.org.uk). RAD loci were inferred from the resulting BAM file of aligned reads, using only read pairs that were properly paired and had a valid CIGAR field. Genome positions with aligned RAD read 1 sequences beginning with a PstI overhang sequence (TGCAG) were labelled as candidate RAD loci. Loci with read 1 sequences aligned in the same direction but mapping to multiple nearby positions within a single read length of 96 bp were discarded, as these loci were probably divergent from the reference genome or incorrectly assembled. Sheared fragment lengths for each read were extracted from the BAM file insert size field, with reads from sheared fragments less than 96 bp long ignored, as these sheared fragments probably contained adapter sequence.

Identical reads at each RAD locus were collapsed to produce a set of unique candidate alleles for the locus. This set of candidate alleles contained real alleles and sequencing errors. Read counts for each allele for each sample were calculated from the alignment. Scaffolds from chromosome 18 only were selected to limit the data set to a manageable size for manual inspection. Parental haplotypes for each individual at each scaffold were known from previous linkage mapping (Heliconius Genome Consortium 2012). For each candidate RAD locus, alleles shared by all individuals with a particular haplotype were associated with the haplotype. Alleles that could not be associated with a haplotype in this way were discarded, filtering out sequencing errors occurring in a small number of individuals. RAD loci were manually inspected to validate assignment of alleles to haplotypes. Many loci with incomplete or inconsistent haplotype assignments were ignored. At RAD loci where a low coverage allele had a number of missing individuals, preventing automatic assignment of the allele to haplotypes by the rule above, but where haplotype assignment were otherwise consistent, the haplotype assignment was manually imputed. Restriction fragment lengths were inferred from the *H. melpomene* genome sequence where RAD loci at both ends of a restriction fragment could be validated. Only restriction fragments contained within single contigs were retained, with restriction fragments spanning multiple contigs within a scaffold discarded, to reduce the risk of including incorrect restriction fragment lengths because of assembly errors.

### Comparison of genotyping tools

Genome Analysis Tool Kit (GATK) v1.6.7, Stacks v0.9992 and RADtools v1.2.2 were used for comparisons. To assess the effect of restriction fragment length skew on read depths, only heterozygous Heliconius genotypes with both alleles present for an individual were included, ignoring missing genotypes and genotypes from RAD loci containing presence/absence calls or indels. All reads aligning to RAD locus positions were included in test read sets, including sequencing errors. For the GATK Unified Genotyper validation, read 2 sequences were not included for genotyping, as Stacks and RADtools both infer loci from read 1 alone. RADtools was run with default options; Stacks was run allowing alleles separated by up to five mismatches to be clustered, to match RADtools defaults. Average read proportions for a pair of restriction fragment lengths were derived by calculating the mean of read proportions for each set of genotypes with a particular pair of restriction fragment lengths (each genotype having two haplotypes and each haplotype having an inferred restriction fragment length). Stacks and RADtools genotypes were linked to known Heliconius RAD loci by allele sequence and considered incorrect if the tools did not cluster the two alleles into one locus, or if the resulting genotype was homozygous rather than heterozygous. GATK base calls were linked to RAD loci by alignment position and considered incorrect if no heterozygous calls were present at any base within the RAD locus.

### RAD contig assembly

Assemblers used for RAD contig assembly comparisons [Fig. 8, Fig. S5 (Supporting Information)] are listed in Table S2 (Supporting Information). Assemblers were run with default parameters unless otherwise specified in the table. Any assembled contig shorter than 100 bases was discarded. Read 2 sets from validated Heliconius PstI loci were used for assembly tests (including sequencing errors, as these reads are typically corrected and included in Stacks and RADtools analyses). Consensus RAD contig sequences for each RAD locus were defined as the maximum region of the *H. melpomene* reference covered by at least one read 2 sequence aligned with Stampy as described earlier. For each assembly, RAD contigs were aligned to the *H. melpomene* genome using BLAST+ v2.2.26, and two metrics were calculated: (i) coverage of the reference by all assembled contigs (sum of bases in the Consensus sequence covered by at least one assembled contig) and (ii) coverage of the reference by the longest assembled contig only.

## Results

### Restriction fragment length biases read depth at RAD loci

To investigate technical variation in RAD-Seq, we constructed a RAD-Seq library using genomic DNA from a pool of *C. elegans* nematode worms (laboratory strain N2) and sequenced the library using an Illumina HiSeq 2000 [see Methods, Table S1 (Supporting Information)]. We used the restriction enzyme PstI, which cuts at the 6 base recognition site CTGCAG, a sequence present at 13,552 locations in the *C. elegans* reference genome. We define a RAD locus as a region up- or downstream of a restriction site covered by read 1 of a read pair (always 96 bp long for the libraries discussed in the main study). We would therefore expect to find two RAD loci for every restriction site, 27,104 in all, if restriction digestion and sequencing are complete. As N2 is a largely homozygous, inbred, matrilineal *C. elegans* strain, RAD loci are expected to be homozygous with few variations from the N2 reference genome. We therefore expect any substantial variation in read depth at RAD loci to be because of the RAD-Seq library preparation procedure rather than RAD loci sequence variation.

**Fig. 1:**
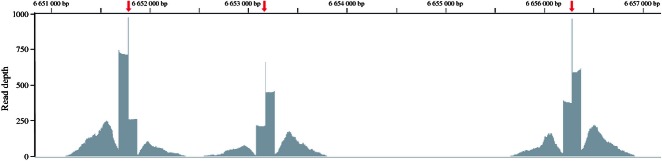
Characteristic pileup of RAD-Seq data. Three PstI restriction sites (red arrows) in *Caenorhabditis elegans* chromosome I (6.651 Mb–6.657 Mb) are covered both upstream and downstream by RAD-Seq raw reads (dark grey). Read 1 sequences are piled up in stacks either side of each restriction site; read 2 sequences are spread out in heaps up to 700 bp beyond the restriction site. The restriction site overhang TGCA is covered by reads belonging to both upstream and downstream RAD loci, producing narrow peaks of read coverage at the restriction sites. RAD loci on either side of a restriction site have different read depths; however, loci from the same restriction fragment have similar read depths. Bases in the read 2 regions are covered at much lower depth overall; read 2 sequences also partially cover the read 1 regions, as seen by the increase in read depth at bases towards the end of read 1 regions.

Restriction site-associated DNA-Seq data have a strikingly different alignment pattern to standard genomic or transcriptomic libraries (Fig. 1; see Thorvaldsdóttir *et al*. 2012 for comparisons). For a set of read pairs aligned to one RAD locus, the beginning of read 1 will align to the restriction site, creating a tall stack of reads directly adjacent to the site, whereas read 2 will be aligned further away from the restriction site, depending on the length of each sheared, sequenced fragment, creating a wide heap of reads covering several hundred bases.

**Fig. 2:**
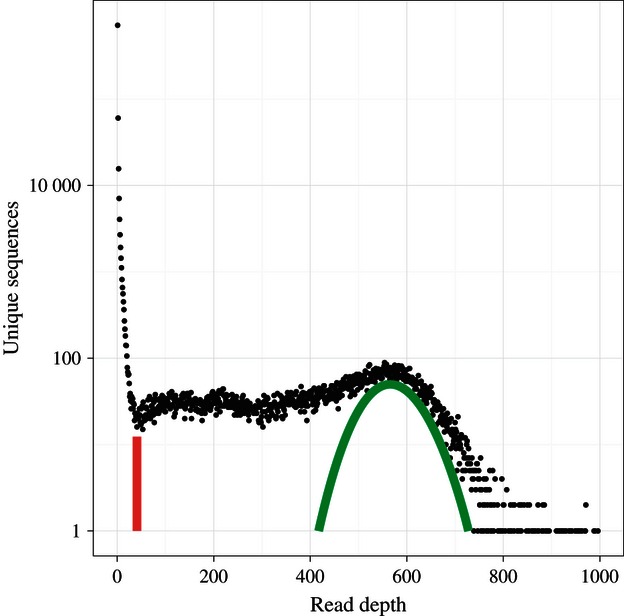
Number of unique 96-bp sequences (*y* axis, log scale) in *C. elegans* PstI RAD-Seq library (14 PCR cycles, Replicate 1) with read depths from 0 to 1000 reads (*x*-axis). An additional long tail of high-count (>1000) unique, repeat-derived sequences is not shown. The red line shows a candidate threshold separating sequencing error from genuine alleles; the green curve shows a theoretical expectation of coverage for homozygous RAD loci.

Our naive expectation was that read depth per RAD locus (the number of read pairs aligning to a locus) would cluster around a single mean with variance approximating a Gaussian distribution because of the high coverage obtained for this library (Fig. 2, green curve; for simulation of RAD data assuming similar conditions, see Catchen *et al*. 2011). However, there is substantial variation in read depth beyond this expectation, as shown at the six RAD loci in Fig. 1 and supported by observed read depths for all unique sequences in the *C. elegans* library (Fig. 2). Sequencing errors (unique sequences with very low read depths) are clearly visible, and a long tail of unique sequences with very high read depths derived from repeats (maximum 55,116 reads) is also present. While the data suggest the presence of a peak representing the bulk of the homozygous alleles, this is obfuscated by a large number of sequences with read depths below this peak, with no separation between sequencing error and these sequences.

**Fig. 3:**
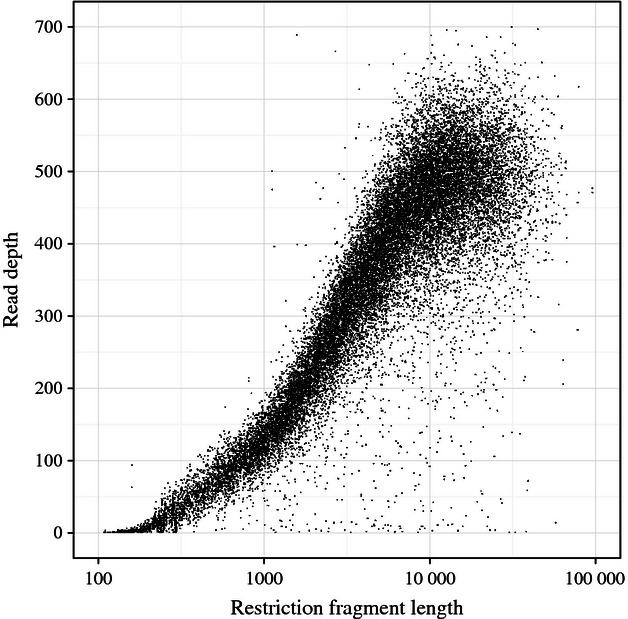
Relationship between restriction fragment length and read depth in *C. elegans* PstI RAD-Seq library (14 PCR cycles, Replicate 1). Only read pairs with perfect read 1 and read 2 alignments within a PstI fragment included in read depths. Repetitive RAD loci or RAD loci with zero read depth excluded.

Restriction cut sites are not evenly distributed across a genome, leading to variation in restriction fragment length. As *C. elegans* has a very high-quality reference genome, it was possible to investigate the relationship between restriction fragment length and read depth for 24,828 unique RAD loci (discarding repeat loci) covered by at least one read (Fig. 3). RAD locus read depth and the logarithm of restriction fragment length are highly correlated (*r*(24 826) = 0.71, *P* < 2.2e-16). This correlation is mostly due to RAD loci from restriction fragments below 10 kb in length. Correlation between read depth and restriction fragment length for RAD loci from restriction fragments longer than 10 kb (6776 loci, 27.2% of all unique covered RAD loci) is significant but very small (*r*(6774) = 0.037, *P* = 0.002); the same correlation for RAD loci from restriction fragments shorter than 10 kb (18,052 loci, 72.7% of loci) is substantial (*r*(18 050) = 0.673, *P* < 2.2e-16). This effect was also detected in other public RAD-Seq data sets (Fig. S1, Supporting Information).

We believe this bias is caused by the shearing step during RAD library preparation. Sonicators are known to shear DNA fragments of different lengths with varying efficiencies (Sambrook & Russell 2006; Berry *et al*. 2012). Plasmids of different lengths shear with varying efficiencies (Fig. S2, Supporting Information), indicating that variation in read depth can be caused by shearing alone, regardless of any other step in the RAD-Seq protocol. In addition, modifying shearing conditions alters the relationship between read depth and restriction fragment length (Fig. S3, Supporting Information). RAD loci from longer restriction fragments have greater read depths whether fragments are sheared using a Covaris sonicator, a Bioruptor or a nebulizer (Fig. S1, Supporting Information), although sonicated shearing and nebulizing produce somewhat different profiles.

### PCR GC content bias is present but minor in RAD-Seq data

**Fig. 4:**
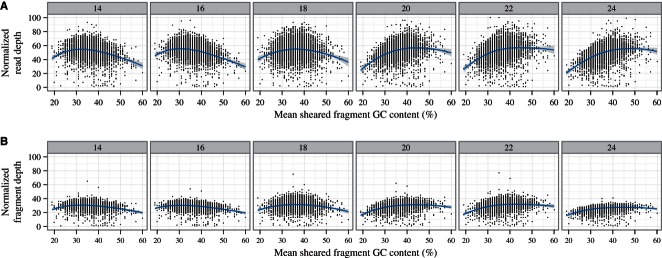
Read depths are influenced by GC content of sheared fragments and number of PCR cycles. PCR cycles vary from 14 (left) to 24 (right) in six separate *C. elegans* samples (Replicate 2 samples used, see Table S1, Supporting Information). *Caenorhabditis elegans* RAD loci with restriction fragment lengths over 10 kb are shown, to minimize restriction fragment length bias. (A) Normalized read depth for mean sheared fragment GC content is shown with Loess curve. (B) Removing PCR duplicates (collapsing multiple copies of unique read pairs) approximates number of sheared fragments and partially corrects PCR bias.

While restriction fragment length explains a large proportion of the variation in RAD-Seq data, it is clear from Fig. 3 that substantial additional variation remains to be explained. As there is a PCR step during the RAD-Seq library preparation, it is expected that the known PCR GC bias in sequencing-by-synthesis data will also be present in RAD-Seq data (Benjamini & Speed 2012; Aird *et al*. 2011; Quail *et al*. 2008). To investigate the effect of PCR amplification, we sequenced six *C. elegans* PstI RAD-Seq samples using 14–24 PCR cycles. PCR biases read depth at RAD loci because of GC content (Fig. 4A), with increasing cycles of PCR causing RAD loci with high GC content to be sequenced at higher depths compared with RAD loci with low GC content. However, this does not necessarily imply that PCR cycles should be minimized; in these samples, high GC content RAD loci appear to be undersequenced at 14 and 16 PCR cycles compared with low GC content RAD loci. This PCR GC bias can be partially alleviated in paired-end RAD-Seq data by removing duplicate read 1 and read 2 pairs, which should approximate the number of DNA fragments in the initial sample (Fig. 4B). We cannot currently account for the remaining variation in RAD-Seq data.

### Restriction site heterozygosity has implications for RAD-Seq genotyping

To investigate the impact of restriction fragment bias on RAD-Seq genotyping, it was necessary to develop a set of heterozygous RAD loci derived from restriction fragments with known lengths. We curated a set of 972 RAD loci from a PstI RAD library of a cross between *Heliconius melpomene melpomene* and *H. m. rosina*, previously used to scaffold the *H. m. melpomene* genome (Heliconius Genome Consortium 2012). RAD loci on scaffolds assigned to chromosome 18 were genotyped, phased and manually curated to remove repeat loci and correct haplotypes that could not be inferred automatically because of missing data in low coverage individuals [see Methods and Additional Discussion (Supporting Information) for further explanation and justification of this data set].

**Fig. 5:**
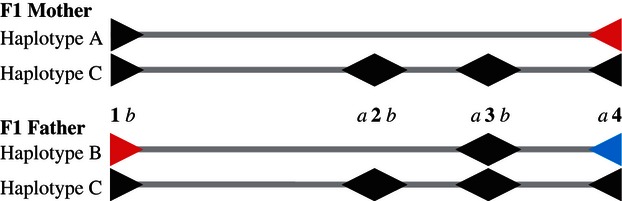
Examples of complex RAD loci. Four consecutive restriction sites are shown (1–4) in each of four haplotypes for two F1 parents, with RAD loci on either side of each site shown as arrowheads and labelled a and b. Haplotype C is shared between the parents. Variant alleles at RAD loci are differentiated by colour (black, red and blue). For example, two variant alleles are found at RAD locus 1b, with the black allele shared between haplotypes A and C and the red allele present in haplotype B only; the black allele has two copies in the mother and one copy in the father. Owing to the heterozygous restriction sites 2 and 3, alleles at RAD loci 1b and 4a are derived from restriction fragments of varying lengths, potentially skewing read depths and resulting in incorrect genotypes. For example, at locus 1b, the father's red allele at Haplotype B will be derived from a long fragment ending at RAD locus 3a, whereas the black allele at Haplotype C will be derived from a shorter fragment ending at RAD locus 2a.

**Fig. 6:**
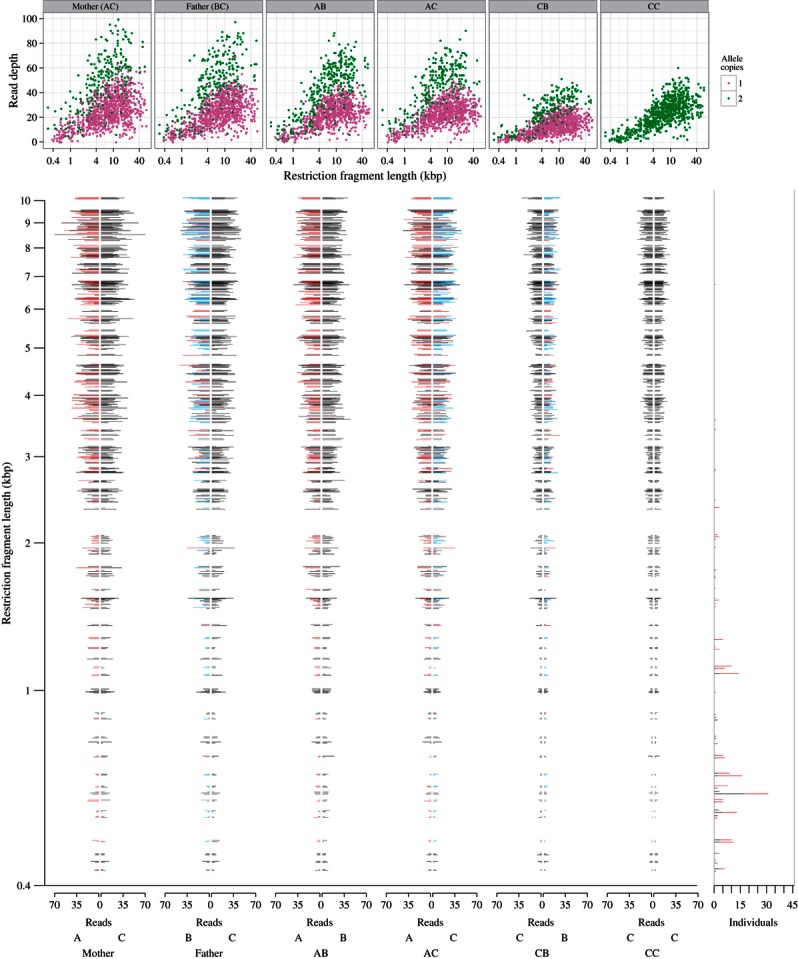
Effect of restriction fragment length bias on missing data and genotyping. Top panel, Heliconius RAD loci exhibit restriction fragment length bias in read depth. Six individuals are shown from the cross: two parents (Mother, genotype AC and Father, genotype BC) and four offspring with each of the four possible F2 genotypes AB, AC, CB and CC (see Fig. 5 for description of haplotypes A, B and C). Individuals vary in overall read depth because of variation in input DNA quantities. Read depths for unique alleles are shown, where allele counts can be 2 (for two-copy alleles at homozygous loci) or 1 (for single-copy alleles at heterozygous loci). Bottom panel, left, read depths for individual allele copies in all individuals vary according to total individual read depth and restriction fragment length. Each RAD locus has two alleles per individual, with read depth per haplotype shown by the length of line extending left or right from centre from 0 to 70 reads. Allele variations at RAD loci are shown in black, red and blue, as per Fig. 5. RAD loci with equal restriction fragment lengths below 10 kb for all haplotypes are shown. Bottom panel, right, genotype accuracy for all 47 individuals in mapping cross for each RAD locus. Black bars, number of individuals with no data for a locus; red bars, number of individuals at heterozygous loci with one of two alleles missing, leading to an incorrect homozygous call.

While in no way representative of a complete genome-wide RAD-Seq data set, this set of loci does allow exploration of several novel features of RAD-Seq data. Figure 5 shows an example of a complex region containing these features; Fig. 6 shows that the set of Heliconius RAD loci have the same restriction fragment bias effect seen in *C. elegans* and that this causes problems for genotyping of RAD loci with low read depths. While alleles present in two copies at homozygous RAD loci have higher read depth overall compared with single-copy RAD alleles from heterozygous loci, read depths for the two sets of alleles overlap, and removing PCR duplicates does not remove this overlap (Fig. S4, Supporting Information).

Older marker development methods based on restriction enzymes focussed on variation in restriction sites, scoring restriction fragment length polymorphisms rather than SNPs (e.g. RFLPs, Botstein *et al*. 1980; AFLPs, Vos *et al*. 1995). Restriction site variation together with the restriction fragment length bias described earlier has novel implications for RAD-Seq data. In a diploid organism, if there is a variation in the restriction site, it may be that one allele will be cut by the restriction enzyme and the other will not (see Fig. 5, sites 2 and 3). For alleles with common restriction fragment lengths, single-copy alleles are present at approximately half the depth of alleles from homozygous RAD loci; however, the restriction fragment length bias described earlier means that, for a single individual, in the absence of restriction fragment length information or genotypes from related individuals, it is not possible to distinguish between a single-copy RAD allele at a RAD locus from a heterozygous restriction site and a long restriction fragment (eg Fig. 5, F1 Father locus 2a), and a two-copy allele at a homozygous RAD locus from a short restriction fragment (eg Fig. 5, F1 Father locus 3b).

**Fig. 7:**
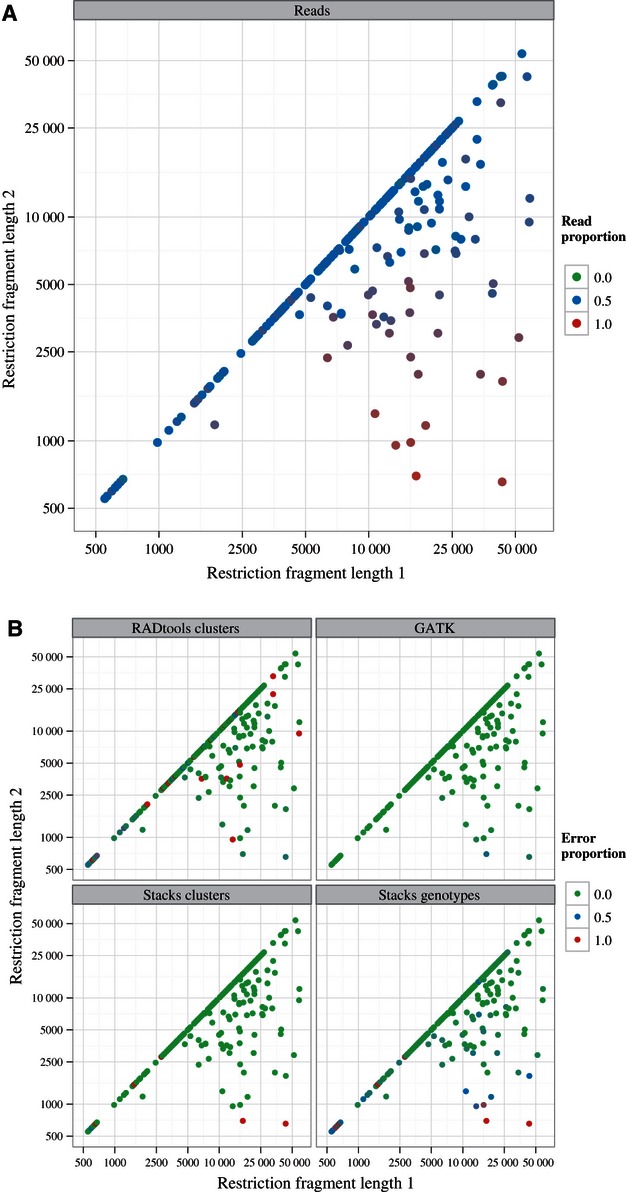
Variable restriction fragment lengths at heterozygous loci skew read depths, which can impact genotyping. Heterozygous genotypes were grouped by restriction fragment lengths. Genotypes where haplotypes have equal restriction fragment lengths are shown on the diagonal; where restriction fragment lengths between haplotypes vary, the longer restriction fragment length is labelled Restriction Fragment Length 1. A, average proportion of read depths for the haplotype with Restriction Fragment Length 1, where 0.5 represents equal read depth for the two haplotypes. B, proportion of incorrect clusterings (RADtools Clusters, Stacks Clusters) or genotypes (Stacks Genotypes, GATK) for all individual genotypes with a particular pair of restriction fragment lengths, with 0.0 indicating perfect calls and 1.0 indicating complete failure to call accurately.

In addition, restriction site heterozygosity combined with restriction fragment read depth bias has implications for genotyping of loci neighbouring a heterozygous restriction site (see Fig. 5, loci 1b and 4a). Alleles from a neighbouring locus may be derived from restriction fragments of different lengths and so have read depths skewed in favour of the longer restriction fragment. There is a clear relationship between restriction fragment length proportion and read depth proportion at heterozygous Heliconius loci (Fig. 7A). This may affect the behaviour of genotypers that assume balanced coverage between alleles at a particular heterozygous locus.

### Effects of restriction fragment length bias and restriction site heterozygosity on genotyping

We processed the 972 Heliconius RAD loci using three software packages commonly used for RAD-Seq analysis, the reference-based GATK Unified Genotyper and the de novo analysis tools Stacks and RADtools (another de novo package, RaPiD, has not been assessed here as it depends on proprietary software that we did not have access to).

Of the 972 RAD loci in the data set, 265 are homozygous, 437 contain SNPs or indels, and 270 are at heterozygous restriction sites and so feature present and absent alleles. Two general features of the genotypers under test cause substantial difficulties. First, Stacks and RADtools are unable to handle indels, meaning that alleles at 314 indel-containing loci are not clustered together. Second, none of the three tools are able to call the absent, uncut alleles at the 270 loci with heterozygous restriction sites; these loci are called as homozygotes for the present, cut allele, not heterozygotes for presence and absence.

This failure to call absent alleles has an additional implication. As RAD loci from short restriction fragments will have low read depths, it is expected that alleles will drop out as restriction fragment length decreases. This can have two effects; either both alleles at a locus drop out for a particular individual, producing no genotype for this individual at this locus, or one of two alleles can drop out; in these cases, the tools call the locus as homozygous rather than heterozygous. Figure 6, bottom right, shows the number of individuals that have one or both alleles dropping out at each RAD locus as restriction fragment length decreases, excluding loci with heterozygous restriction sites.

Figure 7B shows the behaviour of the selected tools in the face of RAD loci with varied restriction fragment lengths and so alleles with skewed read depths, for all heterozygous genotypes. Except for a very small number of failed calls on exceptionally skewed genotypes, GATK is not affected by restriction fragment length skew. In contrast, both Stacks and RADtools fail to cluster alleles and call some genotypes with skewed restriction fragment lengths, although this appears to be because of difficulties calling any genotype with short restriction fragment lengths (as shown by the diagonals, featuring genotypes with equal restriction fragment lengths for both alleles).

### Optimizing RAD-Seq paired-end contig assembly

**Fig. 8:**
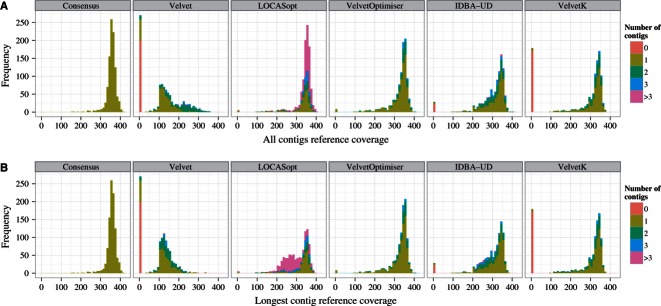
RAD contig assembly comparisons. Consensus panel, maximum possible RAD contig lengths for all Heliconius RAD loci. (A) Number of reference bases covered by all assembled contigs for five assemblers. (B) Number of reference bases covered by longest assembled contig for the same assemblers. Locus assemblies are shown; allele assemblies and additional assembler tests are shown in Fig. S5 (Supporting Information).

We tested nine different assembly tools using the Heliconius RAD loci [Fig. 8, Fig. S5, Table S2 (Supporting Information)]. As alleles were known at these loci, we could group read 2 sequences by locus or by allele and test assembly accordingly. As expected based on previous reports (Baxter *et al*. 2011; Willing *et al*. 2011), unoptimizing assemblers such as Velvet (Zerbino & Birney, 2008), LOCAS (Klein *et al*. 2011) and SOAPdenovo (Li *et al*. 2010) produce poor assemblies, because of considerable variation in coverage and heterozygosity across RAD loci. VelvetOptimiser produces the best assemblies for alleles and loci, but this comes at a very heavy performance cost, taking ∼200x longer than other assemblers (Table S2, Supporting Information). The pre-optimization tool VelvetK goes some way to improving Velvet assemblies without the performance cost, but it fails completely on a subset of loci (Fig. 8); constraining VelvetOptimiser using VelvetK estimates improves this situation but is still not as successful as VelvetOptimiser alone (Fig. S5B,C).

For each locus, Read 2 sequences from all individuals were collapsed together to increase read depth, which is a standard practice but means that the read set contains multiple SNPs. In the face of considerable heterozygosity, assemblers either assemble two full-length contigs varying at SNPs [e.g. VelvetOptimiser, where two contigs are produced for some loci but the longest contig covers the same length as all contigs (Fig. 8B)] or produce multiple contigs broken at SNPs [e.g. LOCASOpt, where the entire Consensus region is usually covered, but many contigs are produced (Fig. 8A), with the longest contig being considerably shorter than the Consensus region length (Fig. 8B)]. These problems are not apparent for allele assemblies, which are homozygous (Fig. S5A, Supporting Information).

## Discussion

### Major factors explaining variation in RAD-Seq data

RAD--Seq is proving to be a highly valuable tool for SNP discovery and genotyping, with many successful applications to date. However, it has been clear for several years that read depths at RAD loci are highly variable. The source of this variation has been unclear, casting doubt on the validity of genotypes at RAD loci and undermining confidence in RAD-Seq analysis methods and results. In this study, we believe we have explained the majority of this variation. The causes of variation will benefit from careful attention and could be handled by more sophisticated data analysis techniques than are currently available. However, in most cases, the variation can be handled by filtering affected loci using simple rules. We believe that RAD markers remaining after filtering can be used with confidence.

The major factor explaining variation in read depth is restriction fragment length bias, caused by incomplete shearing of shorter restriction fragments. As restriction fragment length decreases, efficiency of shearing to a length suitable for Illumina sequencing also decreases, causing unsheared or partially sheared restriction fragments to be discarded on size selection and resulting in a decrease in read depth at affected loci.

A minor version of this effect was expected to affect loci from very short restriction fragments. For example, even if the target sheared fragment size is 500 bp, shearing variation may mean that a single shear of a 1-kb fragment may produce one 800-bp and one 200-bp fragments, both of which would be discarded on size selection to 300–700 bp. This expectation led us to investigate restriction fragment length; however, we did not expect similar shearing inefficiencies to affect restriction fragments up to 10 kb in size, as shown in Fig. 3 and Fig. S1 (Supporting Information). This effect may not severely affect RAD studies using rare cutters but will cause problems for frequent cutters, which are being more frequently used as sequencing costs reduce. This suggests it would be valuable to develop alternative methods of shearing restriction fragments, perhaps using enzymatic protocols such as Nextera or dsDNA fragmentase (Adey *et al*. 2010), or by using additional restriction enzymes (Peterson *et al*. 2012). These methods will require some adaptation to be compatible with RAD-Seq library preparation and will have their own biases, but may avoid the severe restriction fragment bias seen with sonication and, to a lesser extent, nebulization.

Restriction fragment length bias is a locus-specific effect that can cause some RAD loci to be sequenced at very low depths in otherwise well-sequenced libraries (Fig. 6). An additional locus-specific effect, PCR GC content bias, introduces additional variation into RAD-Seq data, similar to that seen in other sequencing-by-synthesis data sets (Fig. 4). While these two effects appear to account for the majority of the variation in RAD-Seq data, sufficient variation remains that it is still not possible to separate alleles by copy number using read depth alone [Fig. 6, top panel; Fig. S4 (Supporting Information)]. There may be additional locus-specific effects that can further explain the remaining variation. However, what remains may be general stochastic variation because of variation in input DNA quantities or accumulated during RAD library preparation.

Restriction site heterozygosity also causes problems for RAD-Seq genotyping. While this behaviour is well known and indeed has been the foundation for traditional marker technologies such as RFLPs and AFLPs, it causes special difficulties for RAD-Seq. First, genotypers designed for whole genome sequencing data do not call absent alleles and mistake heterozygous presence/absence genotypes for homozygous genotypes (Fig. 6). Second, heterozygous RAD loci up- and downstream of a heterozygous restriction site may have skewed read depths because of restriction fragment length bias (Figs 5 and 7).

### Handling special features of RAD-Seq data

While full statistical modelling of the effects described earlier would be desirable (see below), there are simple filters that can be applied to discard most affected RAD loci. First, any RAD locus with a missing genotype could be discarded. This should remove many of the loci with low read depth because of short fragment sizes, because read depths across individuals are usually unbalanced and some individuals may drop out at these sites (Fig. 6, bottom right, black bars). This will also handle many of the heterozygous genotypes where one allele drops out and the genotype is called homozygous, because most of these genotypes occur at loci with some completely missing genotypes (Fig. 6, bottom right; most loci with a red bar also have a black bar). In addition, discarding loci with missing genotypes will filter most of the loci with heterozygous restriction sites, because individuals that are homozygous for the absence of the site at such loci will appear to have a missing genotype (Nadeau *et al*. 2012; Pfender *et al*. 2011).

A more conservative approach to handling the difficulties described earlier would be to discard any locus featuring an allele with restriction fragment length below around 2 kb. In a PstI *C. elegans* library, this would remove 6,254 of 24,826 unique RAD loci or 25% of the data set (ignoring repeats). While this figure is substantial, over 18,000 loci remain, a large data set for many applications, and these loci can be reliably genotyped by the tools tested here (Fig. 7B).

This conservative approach will be overly cautious for many applications. If a reference genome of reasonable quality is available, the GATK Unified Genotyper should be able to call accurate genotypes at almost all loci, even those with severely skewed read depths. But this filtering may be desirable for de novo applications, as both Stacks and RADtools have difficulty calling genotypes accurately for low coverage loci, even where there are no missing genotypes, whether restriction fragment lengths are skewed or not (Fig. 7B). However, as a reference genome is unavailable for de novo analyses, restriction fragment lengths are unknown, and so a read depth threshold must be used as a proxy. This threshold could be estimated by plotting read depth of unique sequences as per Fig. 2, estimating the read depth at the peak (shown in green in Fig. 2), and crudely filtering by discarding any locus with approximately a third of this read depth or less. This analysis should be performed per individual as read depths across individuals can vary considerably.

These crude, conservative approaches will discard many RAD loci that could be rescued with more sophisticated analysis. First, it may be possible to genotype RAD loci at heterozygous restriction sites accurately based on existing genotype calls. GATK produces lower quality scores for single-copy alleles than for two-copy alleles at these loci, which may allow single-copy alleles to be identified by modelling of quality scores. Second, although read depth alone is not enough to estimate allele copy number in the absence of additional contextual information [Fig. 6, top panel, Fig. S4 (Supporting Information)], it may be possible to build a statistical model that would generate likelihoods for each allele copy number for each genotype at each locus.

Restriction site-associated DNA-Seq read depths are count data, which are traditionally modelled with a Poisson or negative binomial distribution, but these distributions do not fit RAD data well because of restriction fragment length bias. However, the relationship of the logarithm of restriction fragment length to read depth appears to closely follow a generalized logistic curve (Fig. 3); if this curve could be modelled and controlled for, variation for RAD loci with a particular restriction fragment length may follow a Poisson or negative binomial distribution as for simple count data. It would therefore be possible to build a hierarchical model to estimate the asymptote of read depth in each sample in the library and estimate the likelihoods of an individual genotype featuring one or two copies of an allele, given the other genotypes at the same locus and the set of sample asymptotes. In addition, it may be possible to incorporate PCR GC content bias into this model. This approach is unlikely to be required for simple applications where other contextual information is available, but it may be important for complex applications involving wild populations.

### Tools for RAD-Seq analysis

We show here the benefits of optimizing assembly parameters for RAD contig assembly, because of large variation in read depth and complexity of RAD loci (Figs 8, S5), in agreement with the study by Willing *et al*. (2011). VelvetOptimiser produces the highest quality assemblies, but the performance cost is severe (Table S2, Supporting Information), and high throughput use will probably require a compute cluster. It is important to note that assemblers run with default options (e.g. Velvet, SOAPdenovo) produce very poor assemblies; it is essential to estimate assembly parameters per locus to achieve a good result. However, it may be possible to derive metrics to estimate suitable Velvet parameters directly without carrying out multiple assemblies, as is done by VelvetOptimiser (see Etter *et al*. 2011b). IDBA-UD (Peng *et a*l. 2012) produces good-quality assemblies for most loci with a fraction of the performance cost 1 of VelvetOptimiser and so may be a suitable alternative.

We have not undertaken a full assessment of RAD-Seq analysis tools as we had no appropriate validated data set available for comparison. We believe the Heliconius data set used here is suitable for exploring the effects of restriction fragment length and restriction site heterozygosity, but that it underestimates the complexity of real RAD data sets and so was not suitable for further assessments (see Additional Discussion, Supporting Information). No doubt substantial problems remain with calling repeat loci, and it may be that other flaws in analysis tools remain undetected. However, we see no serious cause for concern based on the results presented here regarding GATK, Stacks or RADtools. We present RADtools here as it has been used for several other projects, including some presented in this issue (e.g. Richards *et al*. This Issue); while we see no serious reason to question these results, we recommend Stacks for future projects, as it has many additional features, more sophisticated genotyping and much better performance.

In conclusion, we believe that barring the issues discussed earlier, RAD loci cause no additional difficulties to existing tools that are not already widely known from whole genome sequencing projects. For example, handling low depth or repeat loci are issues widely discussed elsewhere in the literature (DePristo *et al*. 2011; Nielsen *et al*. 2011), and missing alleles will also occur with structural variation (Alkan *et al*. 2011). Therefore, we believe that once the issues discussed here have been avoided or handled, genotypes from RAD loci can be used with confidence.

## Data accessibility

Heliconius RAD data are available under European Nucleotide Archive project ERP000993. *Caenorhabditis elegans* RAD samples described in Table S1, MiSeq data for Fig. S3 and Heliconius PstI RAD reads aligned to used chromosome 18 scaffolds are available under European Nucleotide Archive project ERP001757. Other data files and custom Perl, Python and R scripts used for data analysis are available at Dryad under DOI 10.5061/dryad.218p2.

## Supporting information

**Appendix S1** Additional methods

**Fig. S1** Effect of restriction fragment length in three RAD-Seq data sets.

**Fig. S2** Shearing of plasmids pOJ260 (3.5 kb, red) and pLUM (9 kb, blue).

**Fig. S3** Read depth varies with modified shearing conditions.

**Fig. S4** *Heliconius* allele counts cannot be separated by removing PCR duplicates.

**Fig. S5** Additional RAD contig assembly comparisons.

**Table S1** *Caenorhabditis elegans* PstI RAD library details.

**Table S2** Restriction site-associated DNA contig assembly details.

**Table S3** Restriction site-associated DNA adapter and primer sequences.
