# Supplementary material for: Special features of RAD Sequencing data: implications for genotyping
Source: Mol Ecol. 2012 Oct 30;22(11):3151–64. doi: 10.1111/mec.12084 (PMC3712469; doi:10.1111/mec.12084)
Supplement: Supplementary file 1 [file mec0022-3151-SD1.pdf]

# Special features of RAD Sequencing data: implications for genotyping

John W. Davey, Timothée Cezard, Pablo Fuentes Utrilla, Cathlene Eland, Karim Gharbi, Mark L. Blaxter

# Supporting Information

## Additional Methods

### *Plasmid digestion and shearing*

To evaluate the effect of fragment length on shearing efficiency (Figure S2) two commercial cloning vectors of different sequence lengths (pOJ260, 3,469 bp, GenBank GU270843.1, and pcDNA3 RL-GW, 7,981 bp, www.dkfz.de/gpcf/749.html), were subjected to the equivalent shearing conditions used in RAD-Seq library preparation. Cloning vectors were linearized by digesting 2.5 μg of each vector with 20 U of EcoRI-HF (New England BioLabs) in a 50 μL reaction using two replicates per vector. Digestions were diluted with water to a volume of 135 μL, with 130 μL transferred to a Covaris microTUBE with AFA fiber for shearing in a Covaris S2. Shearing conditions used followed the manufacturer's recommended values for 500 bp target peak (www.covarisinc.com, protocol pn_40056). Pre- and post-shearing sample DNA fragments were analyzed by microfluidic capillary electrophoresis (High Sensitivity DNA Chip Assay in an Agilent 2100 Bioanalyzer, Agilent Technologies).

### *C. elegans libraries for shearing test*

Two additional PstI RAD libraries were prepared to investigate the impact of shearing on read depth (Figure S3). Short RAD adapters were used for these libraries as per Andersen et al. (2012), modified for paired end sequencing and indexing on the Illumina platform (Table S3). Both libraries were prepared with 4.8 μg gDNA and enriched with different indexing P2 PCR primers (three per library). The libraries were subjected to two different shearing conditions in a Covaris S2 sonicator using TC13 tubes (max. 1.5 mL sample). In library "Single", all DNA was sheared to 10 shearing cycles, one shearing cycle consisting of a shearing step

(duty cycle 20%, intensity 5, 200 cycles per burst, cycling time 30s) and a *pause* step to allow cooling of the sample (duty cycle 3%, intensity 1; 50 cycles per burst, cycle time 20s). In library "Mixed", one half of the sample DNA was sheared to 10 cycles and the other half to 20 cycles. After 14 cycles of PCR enrichment, libraries were normalized to 15nM, pooled together equimolarly and sequenced in one Illumina MiSeq run (150 bp paired end reads).

### *Sequence analysis for additional public RAD libraries*

Two additional public data sets are shown in Figure S1: EcoRI *C. elegans* RAD data (Andersen *et al.* 2012, Sequence Read Archive accession 047839, library ECA17, kindly made available by E. Andersen and J. Shapiro before SRA release) and SbfI stickleback RAD data (Hohenlohe *et al.* 2010, Sequence Read Archive run SRR034312, aligned to *G. aculeatus* genome assembly BROAD S1 in Ensembl release 66). These data sets were analysed as for Figure 3, described in the main Methods section, except that as only read 1 data was available for the EcoRI and SbfI samples, our *C. elegans* PstI data was reanalysed using only read 1 data. Libraries were subsampled for comparison to 6,516,557 reads, the number of uniquely mapping reads in the selected stickleback library.

### *Data analysis and graphics tools*

Loess regression curves and all plots generated with ggplot version 0.9.0 (Wickham 2009), except Figure 1, produced using IGV v2.1.16 (Thorvaldsdóttir *et al*. 2012), and the lower panels of Figure 6, produced using the R grid package (Murrell 2005). Figures and tables were prepared for publication using Adobe Illustrator v5.1 and Apple iWork '09.

# Additional Discussion

*Test data for RAD analyses*

To test the biases described here, it was necessary to develop a set of heterozygous RAD loci with known restriction fragment lengths. We have used a RAD library from a *Heliconius* mapping cross for this purpose. This data was the only publicly available data known to us that featured a reference genome (allowing measurement of restriction fragment lengths), was highly heterozygous (allowing tests of many special features of RAD data as shown in Figure 5), had known, simple genetic structure (making phasing of haplotypes possible, essential for testing alleles with varying restriction fragment lengths) and had paired end reads available for assessment of RAD contig assembly.

However, this data set has several drawbacks. Firstly, the *H. melpomene* reference genome is new and, as with all recent genome sequences, has many errors. We have attempted to avoid many of these by only including restriction fragments contained within a single contig, and discarding those within a scaffold but spanning multiple contigs. This has limited the available fragment lengths to some extent, with few RAD loci having fragment lengths over 10 kb. Secondly, we have no external data set (for example, whole genome sequencing) to compare the RAD genotypes to, and so the validated genotypes are entirely derived from the RAD data itself. We have confidence in our identification of recombinants in this family, and so in our genotypes for each individual, making it possible to have some confidence in genotypes across loci and in our inference of genotypes for missing data. But many loci were excluded in this process; the data set is intended to be free of repetitive loci and loci with questionable genotypes were excluded.

This means that, while this data set was suitable for exploring the effects of restriction fragment length and restriction site heterozygosity, it underestimates the complexity of real RAD data sets, and so was not suitable for a full assessment of the tools discussed here. It would be highly desirable to develop a data set of heterozygous loci for a species with a reference genome where genotypes are already known from other data sources. This data set could be used to more thoroughly validate existing tools and search for other biases in RAD data that may not have been detected here.

# Legends for Supporting Figures and Tables

**Figure S1** Effect of restriction fragment length in three RAD-Seq data sets. Left panels, relationship of restriction fragment length to read depth as per Figure 3. Right panels, histograms showing frequency of restriction fragment lengths for each restriction enzyme in the relevant reference genome, indepedent of the RAD libraries. All three libraries show a tendency for read depth to increase as restriction fragment length increases, until fragment lengths reach 10 kb, a boundary clearly visible in **A** and **C** but not reached in **B** due to lack of long fragments produced by EcoRI. Density of points reflects number of restriction cut sites (PstI, 13,552; EcoRI, 45,939; SbfI, 19,354). Heterozygosity of sample has a large influence on read depth profile; the wild samples shown in **C** are more comparable to the *Heliconius* data shown in Figure 6, top panel, than the clonal lab strain shown in **A**.

**Figure S2** Shearing of plasmids pOJ260 (3.5 kb, red) and pLUM (9 kb, blue). Top panel, unsheared; bottom panel, sheared. Neither of the plasmids is completely sheared, but shearing of the 3.5 kb plasmid is less complete than the 9 kb plasmid.

**Figure S3** Read depth varies with modified shearing conditions. Two *C. elegans* PstI RAD libraries were sequenced. The first was sheared as per the other *C. elegans* libraries, using 10 shearing cycles (Single). The second was a pool of two libraries, one sheared using 10 cycles, another sheared using 20 cycles (Mixed). Additional shearing increases read depth for RAD loci from short restriction fragments and proportionally reduces depth for RAD loci from longer restriction fragments. Further experimentation with varied shearing of additional aliquots may permit balancing of read depth across the range of fragment lengths.

**Figure S4** *Heliconius* allele counts can not be separated by removing PCR duplicates. The same loci as shown in Figure 6, top panel, are shown with Sheared Fragment Depth rather than Read Depth. While variance is reduced, there is still an overlap between depth of single copy alleles and two copy alleles.

**Figure S5** Additional RAD contig assembly comparisons. **A**, assembly by allele rather than locus avoids difficulties with heterozygosity. **B** and **C**, coverage for all contigs (**B**) and longest contig (**C**) for four additional assemblers. VelvetK and VelvetOptimiser together approach the quality of VelvetOptimiser alone but some failed assemblies remain (red bar on left).

**Table S1** *C. elegans* PstI RAD library details. Four replicates for each test of 14, 16, 18, 20, 22 and 24 PCR cycles were pooled together using 24 different barcodes. Fragments are unique read1+read2 sequence counts, approximating the number of DNA fragments in initial samples after removal of PCR duplicates. Proportions calculated as described in Materials and methods.

**Table S2** RAD contig assembly details. URLs are given where no publication exists for an assembly tool. While optimized assemblers perform considerably better than unoptimized assemblers, the performance cost is substantial.

**Table S3** RAD-Seq adapter and primer sequences. Adapter oligos were ordered from Integrated DNA Technologies (Ultramer synthesis, TruGrade purification). PCR primers were ordered from Sigma-Aldrich (PAGE purified).

# Figure S1

**A** *C. elegans* N2 lab strain cut with PstI, sheared with Covaris sonication

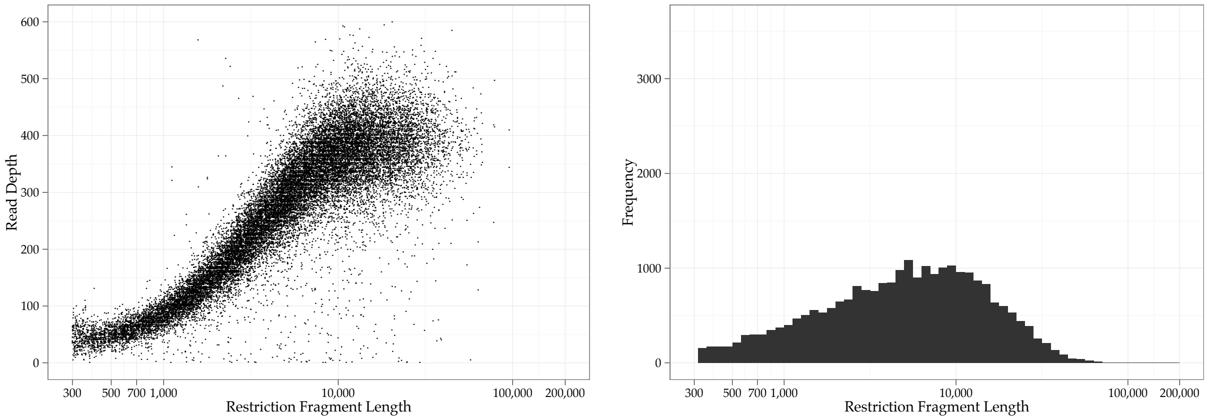


**B** *C. elegans* N2-like wild strains cut with EcoRI, sheared with nebulization (Andersen *et al.* 2012)

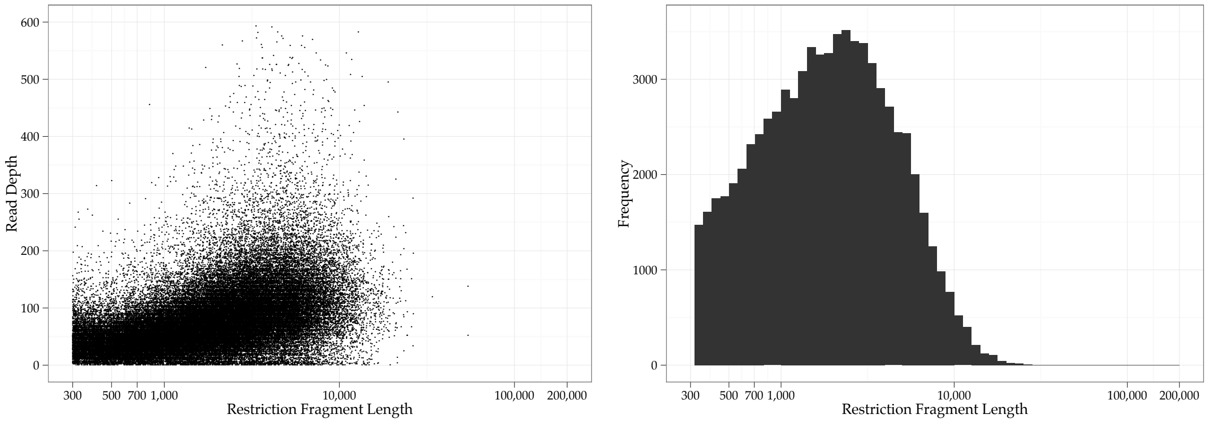


**C** *G. aculeatus* wild population cut with SbfI, sheared with Bioruptor sonication (Hohenlohe *et al.* 2010)

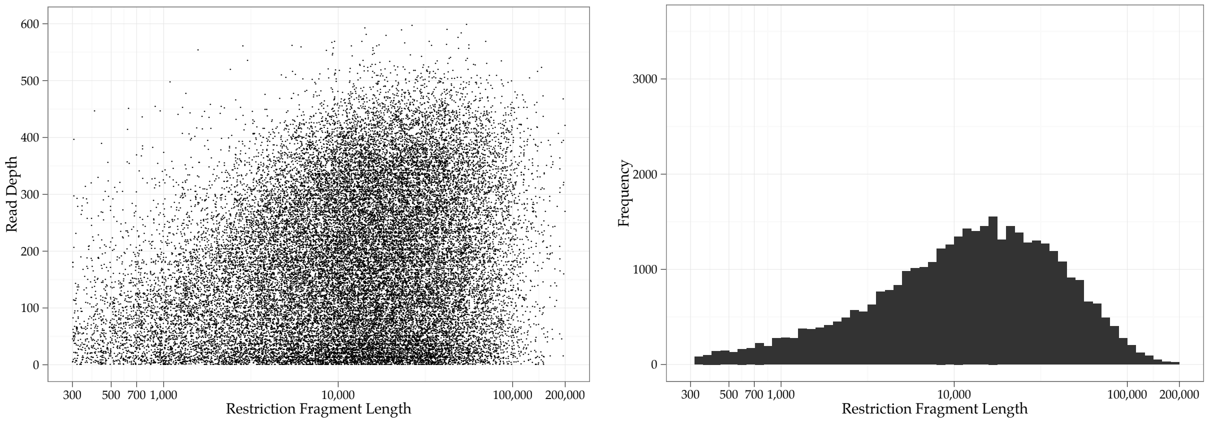

# Figure S2

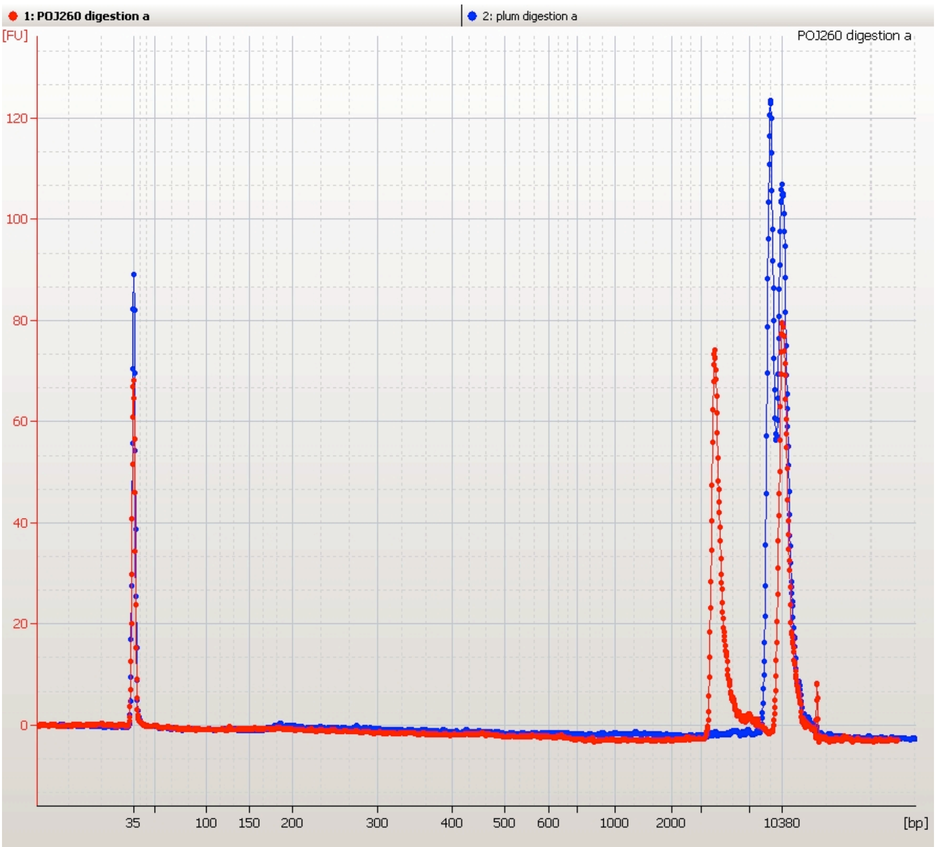
1: POJ260 digestion a
2: plum digestion a
POJ260 digestion a
[FU]
120
100
80
60
40
20
0
35
100
150
200
300
400
500
600
1000
2000
10380
[bp]

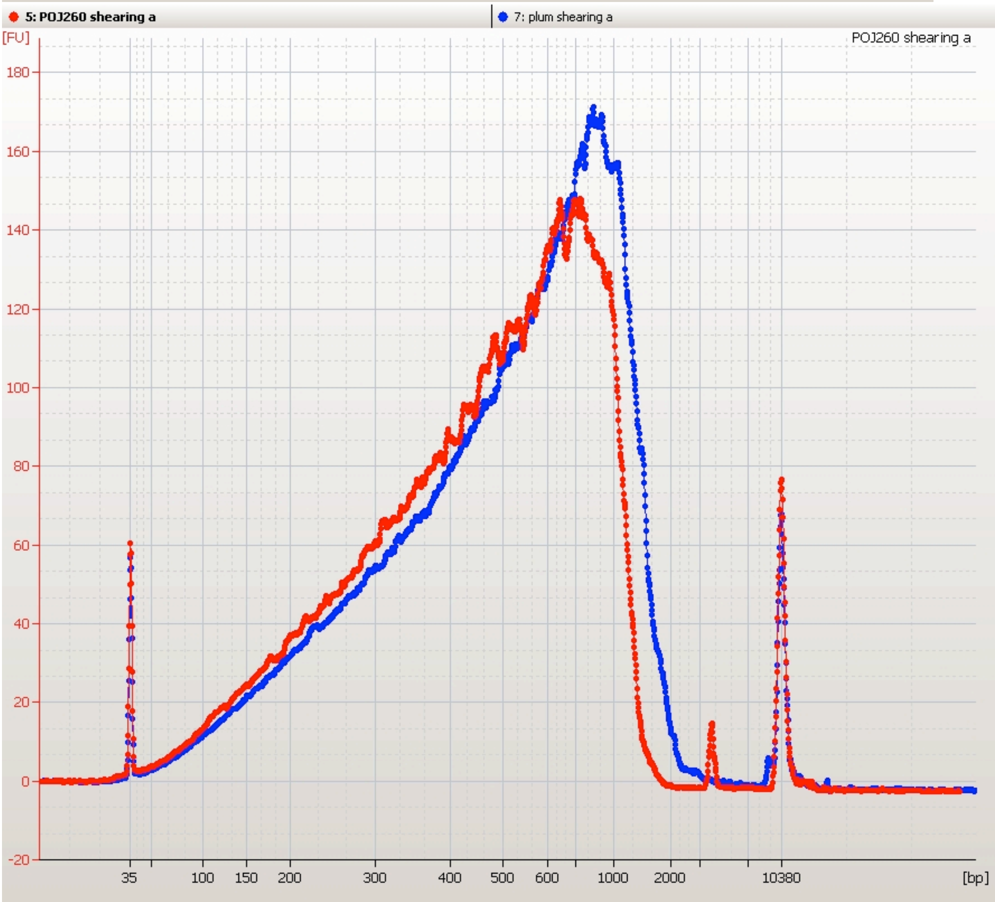
5: POJ260 shearing a
7: plum shearing a
POJ260 shearing a
[FU]
180
160
140
120
100
80
60
40
20
0
-20
35
100
150
200
300
400
500
600
1000
2000
10380
[bp]

# Figure S3

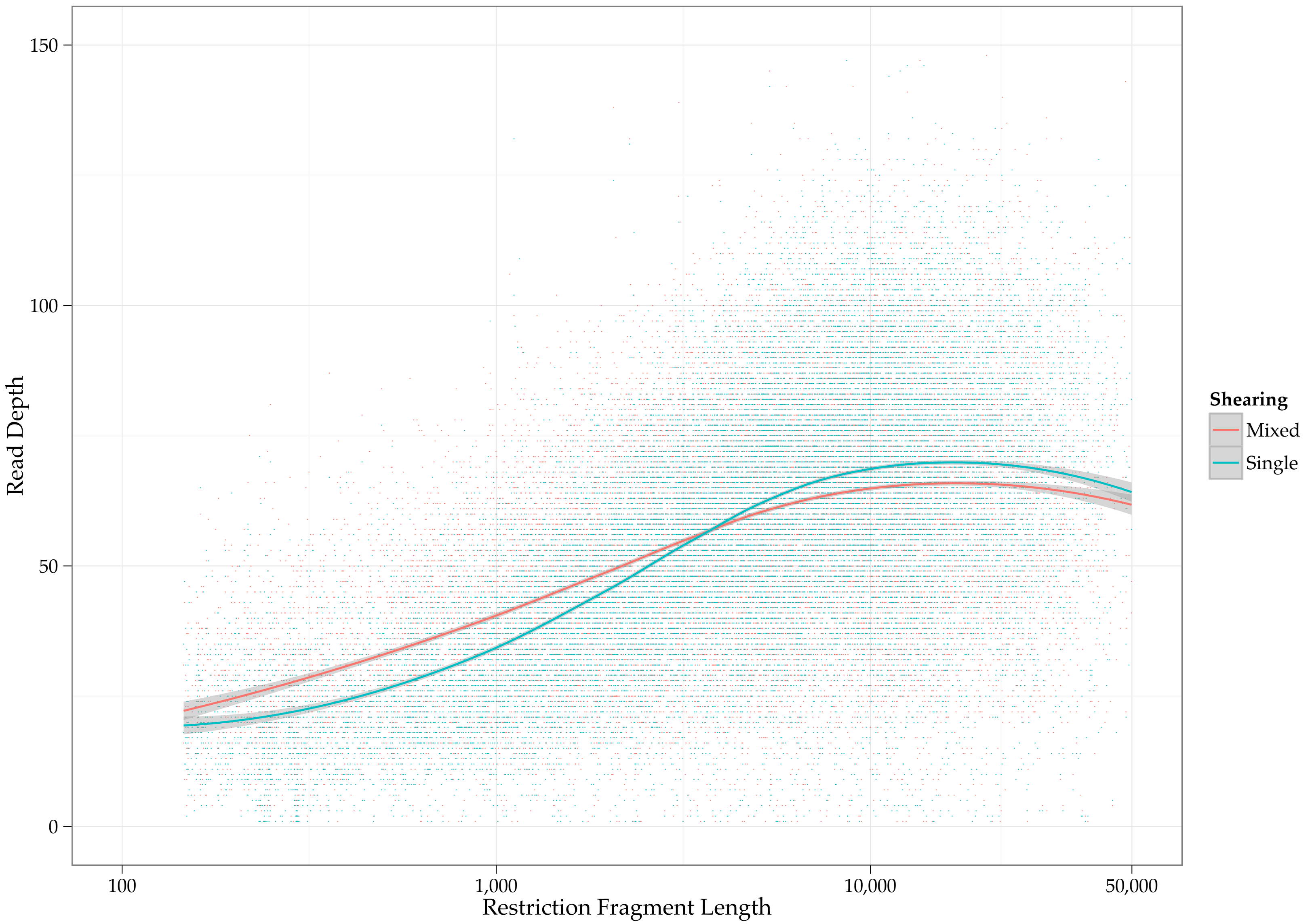

150
100
50
0
Read Depth
100
1,000
10,000
50,000
Restriction Fragment Length
Shearing
Mixed
Single

# Figure S4

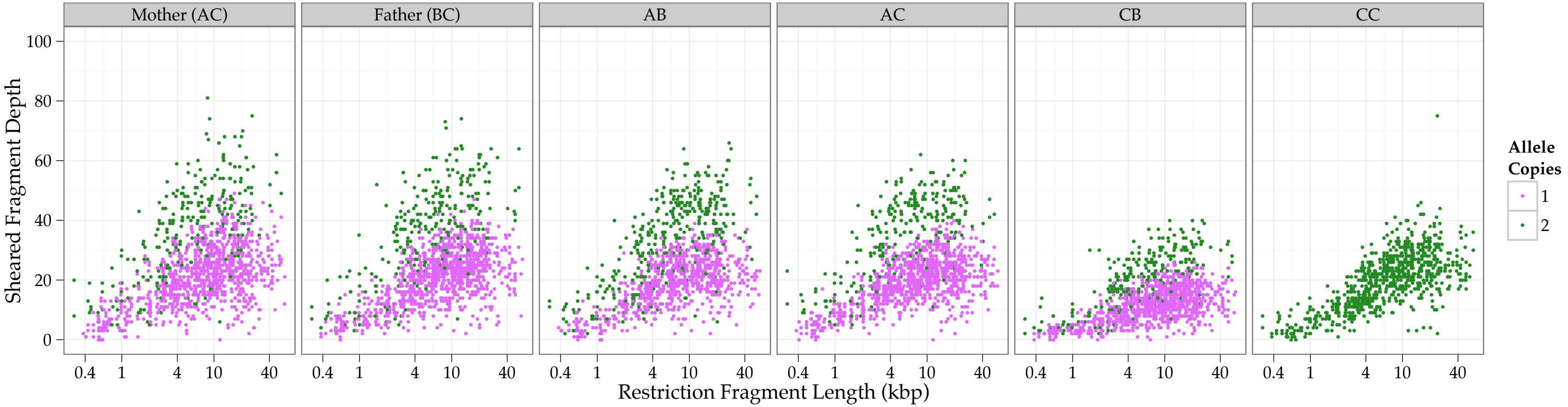

Mother (AC)
Father (BC)
AB
AC
CB
CC
Sheared Fragment Depth
0
20
40
60
80
100
0.4
1
4
10
40
Restriction Fragment Length (kbp)
Allele Copies
1
2

# Figure S5

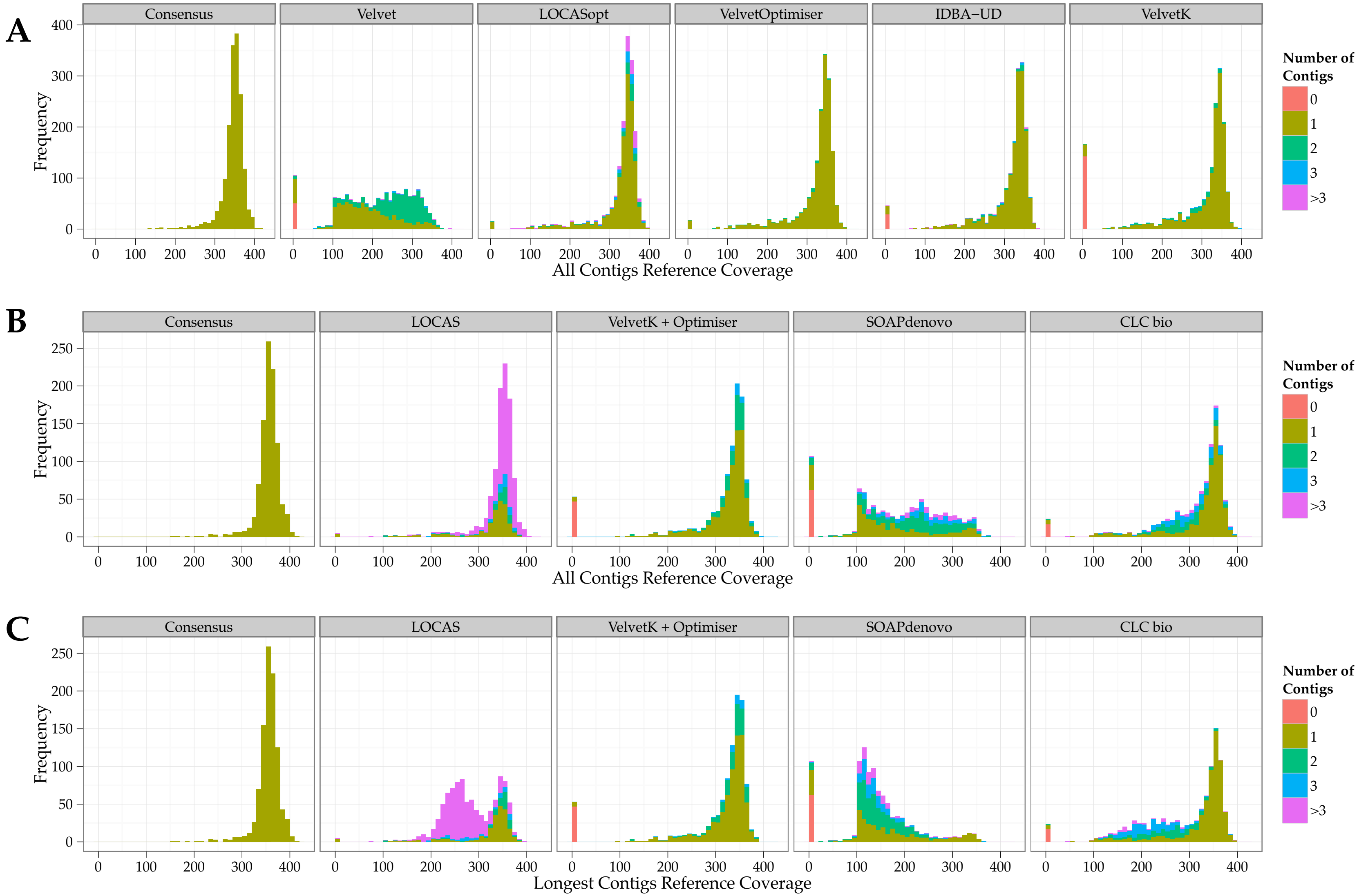
A
B
C
Consensus
Velvet
LOCASopt
VelvetOptimiser
IDBA−UD
VelvetK
LOCAS
VelvetK + Optimiser
SOAPdenovo
CLC bio
Frequency
All Contigs Reference Coverage
Longest Contigs Reference Coverage
Number of Contigs
0
1
2
3
>3

# Table S1

| PCR cycles | Replicate | Barcode | Reads | Perfectly Aligned Reads | Read Proportion | Perfectly Aligned Fragments | Fragment Proportion |
|---|---|---|---|---|---|---|---|
| 14 | 1 | ACCAT | 12,326,499 | 8,379,012 | 9.59 | 4,304,560 | 8.29 |
| | 2 | CTAGG | 3,948,749 | 2,599,743 | 2.98 | 1,845,053 | 3.55 |
| | 3 | GGTTC | 3,858,053 | 2,584,773 | 2.96 | 1,853,492 | 3.57 |
| | 4 | TAGCA | 7,245,703 | 4,820,991 | 5.52 | 2,963,020 | 5.70 |
| 16 | 1 | AGTCA | 3,074,799 | 2,049,193 | 2.35 | 1,380,758 | 2.66 |
| | 2 | CGCGC | 6,425,852 | 4,289,467 | 4.91 | 2,536,337 | 4.88 |
| | 3 | GATCG | 4,402,052 | 2,911,895 | 3.33 | 1,852,804 | 3.57 |
| | 4 | TTAAT | 3,322,388 | 2,199,939 | 2.52 | 1,443,247 | 2.78 |
| 18 | 1 | ACTGC | 11,077,912 | 7,058,494 | 8.08 | 3,594,563 | 6.92 |
| | 2 | CGTAT | 1,500,152 | 936,050 | 1.07 | 757,827 | 1.46 |
| | 3 | GTACA | 3,840,509 | 2,394,621 | 2.74 | 1,669,857 | 3.21 |
| | 4 | TGTGG | 2,296,297 | 1,434,825 | 1.64 | 1,101,822 | 2.12 |
| 20 | 1 | ATCGA | 2,030,189 | 1,292,540 | 1.48 | 1,093,084 | 2.10 |
| | 2 | CAGTC | 4,576,312 | 2,880,937 | 3.30 | 2,141,614 | 4.12 |
| | 3 | GCATT | 4,161,704 | 2,623,745 | 3.00 | 1,984,393 | 3.82 |
| | 4 | TCGAG | 4,659,716 | 2,946,855 | 3.37 | 2,181,666 | 4.20 |
| 22 | 1 | ATATC | 7,350,846 | 4,693,005 | 5.37 | 2,731,100 | 5.26 |
| | 2 | CACAG | 2,705,832 | 1,704,638 | 1.95 | 1,196,171 | 2.30 |
| | 3 | GGCCT | 3,640,740 | 2,307,852 | 2.64 | 1,546,958 | 2.98 |
| | 4 | TCAGA | 7,603,924 | 4,849,554 | 5.55 | 2,778,670 | 5.35 |
| 24 | 1 | AGAGT | 11,324,477 | 7,565,595 | 8.66 | 3,696,726 | 7.12 |
| | 2 | CCAAC | 16,077,734 | 10,842,389 | 12.41 | 4,640,207 | 8.93 |
| | 3 | GACTA | 2,367,316 | 1,545,522 | 1.77 | 1,058,500 | 2.04 |
| | 4 | TTCCG | 3,711,388 | 2,453,537 | 2.81 | 1,589,064 | 3.06 |

# Table S2

| Assembler | Version | Reference | Parameters | Mean time per assembly (s) | |
|---|---|---|---|---|---|
| | | | | By locus | By allele |
| **CLCbio** | 4.06beta.67189 | http://www.clcbio.com | Default | 0.46 | 0.25 |
| **IDBA-UD** | 1.0.9 | Peng *et al.* 2012 | Default | 1.49 | 0.63 |
| **LOCAS** | 0.1.7 | Klein *et al.* 2011 | Default | 2.85 | 0.48 |
| **LOCASOpt** | 0.5 | Willing *et al.* 2011 | kmer range 11-15<br>required overlap length 21-35 bases,<br>error rate 0.02-0.04 | 87.20 | 18.75 |
| **SOAPdenovo** | 1.05 | Li, Zhu *et al.* 2010 | Default | 0.26 | 0.13 |
| **Velvet** | 1.2.05 | Zerbino *et al.* 2008 | kmer 29 | 0.74 | 0.24 |
| **VelvetK** | 6 June 2012 | http://www.vicbioinformatics.com/software.velvetk.shtml | use --best option<br>then run Velvet with kmer chosen by VelvetK | 0.91 | 0.63 |
| **VelvetOptimiser** | 2.2.0 | http://www.vicbioinformatics.com/software.velvetoptimiser.shtml | kmer range 19-99<br>optimise for longest contig | 170.58 | 170.62 |
| **VelvetK + VelvetOptimiser** | As above | As above | VelvetOptimiser kmer range +/-10 from VelvetK estimate | 46.57 | 46.40 |

# Table S3

| Name | Sequence | Notes |
|---|---|---|
| **Short P1 Adapter Top** | ACACTCTTTCCCTACACGACGCTCTTCCGATCT**XXXXXXXX**TGC*A | **XXXXXXXX** = 8bp barcode sequence;<br>* = phosphothiorate bond |
| **Short P1 Adapter Bottom** | /5Phos/**YYYYYYYY**AGATCGGAAGAGCGTCGTGTAGGGAAAGAGTGT | **YYYYYYYY** = 8bp barcode sequence, reverse complement; 5Phos = added phosphate |
| **Short P2 Adapter Top** | /5Phos/GATCGGAAGAGCGGTTCAGCAGGTCCATTC | 5Phos = added phosphate |
| **Short P2 Adapter Bottom** | GAGATCGGTCTCGGCATTCCTGCTGAACCGCTCTTCCGATCT*T | * = phosphothiorate bond |
| **Forward amplification primer** | AATGATACGGCGACCACCGAGATCTACACTCTTTCCCTACACGACGCTC*T | * = phosphothiorate bond |
| **Reverse amplification primer** | Sequence provided by Michael A Quail (mq1@sanger.ac.uk; Wellcome Trust Sanger Institute, Hinxton, Cambridge, CB10 1SA, UK), available on request | |
